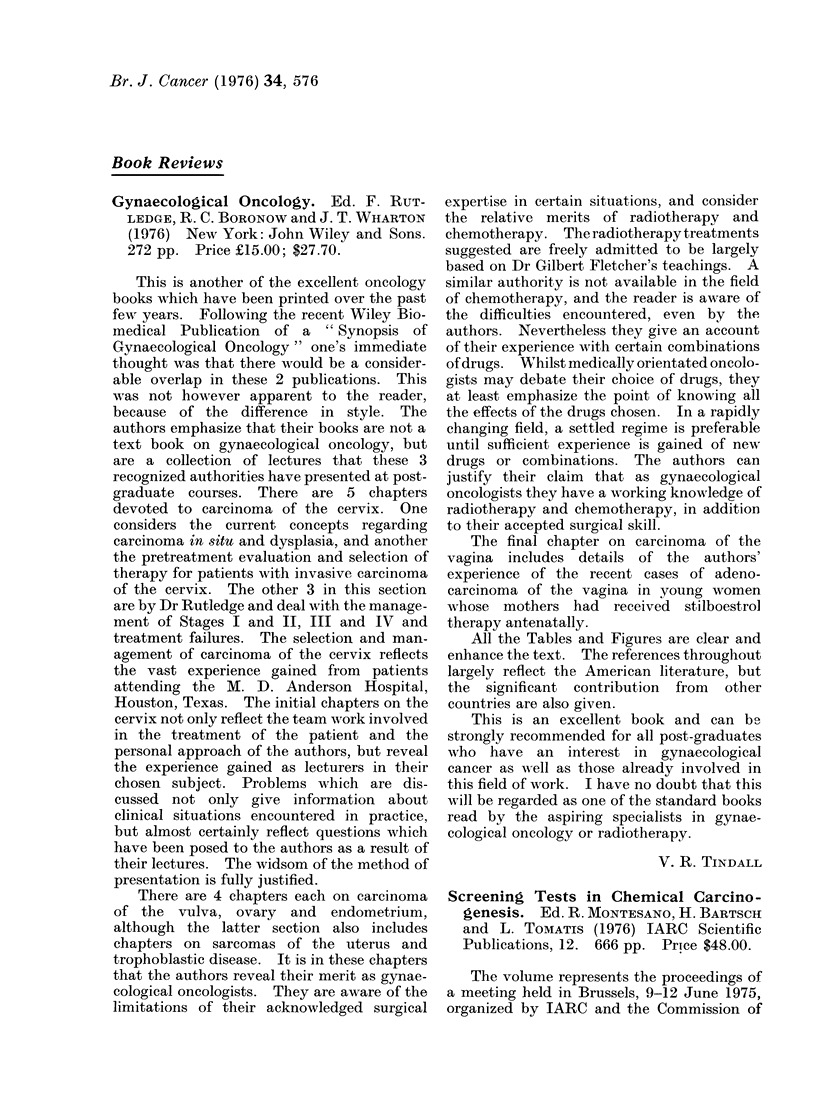# Gynaecological Oncology

**Published:** 1976-11

**Authors:** V. R. Tindall


### *Book Reviews*

**Gynaecological Oncology.** Ed. F. Rutledge, R. C. Boronow and J. T. Wharton (1976) New York: John Wiley and Sons. 272 pp. Price £15.00; $27.70.

This is another of the excellent oncology books which have been printed over the past few years. Following the recent Wiley Biomedical Publication of a " Synopsis of Gynaecological Oncology " one's immediate thought was that there would be a considerable overlap in these 2 publications. This was not however apparent to the reader, because of the difference in style. The authors emphasize that their books are not a text book on gynaecological oncology, but are a collection of lectures that these 3 recognized authorities have presented at postgraduate courses. There are 5 chapters devoted to carcinoma of the cervix. One considers the current concepts regarding carcinoma *in situ* and dysplasia, and another the pretreatment evaluation and selection of therapy for patients with invasive carcinoma of the cervix. The other 3 in this section are by Dr Rutledge and deal with the management of Stages I and II, III and IV and treatment failures. The selection and management of carcinoma of the cervix reflects the vast experience gained from patients attending the M. D. Anderson Hospital, Houston, Texas. The initial chapters on the cervix not only reflect the team work involved in the treatment of the patient and the personal approach of the authors, but reveal the experience gained as lecturers in their chosen subject. Problems which are discussed not only give information about clinical situations encountered in practice, but almost certainly reflect questions which have been posed to the authors as a result of their lectures. The widsom of the method of presentation is fully justified.

There are 4 chapters each on carcinoma of the vulva, ovary and endometrium, although the latter section also includes chapters on sarcomas of the uterus and trophoblastic disease. It is in these chapters that the authors reveal their merit as gynaecological oncologists. They are aware of the limitations of their acknowledged surgical expertise in certain situations, and consider the relative merits of radiotherapy and chemotherapy. The radiotherapy treatments suggested are freely admitted to be largely based on Dr Gilbert Fletcher's teachings. A similar authority is not available in the field of chemotherapy, and the reader is aware of the difficulties encountered, even by the authors. Nevertheless they give an account of their experience with certain combinations of drugs. Whilst medically orientated oncologists may debate their choice of drugs, they at least emphasize the point of knowing all the effects of the drugs chosen. In a rapidly changing field, a settled regime is preferable until sufficient experience is gained of new drugs or combinations. The authors can justify their claim that as gynaecological oncologists they have a working knowledge of radiotherapy and chemotherapy, in addition to their accepted surgical skill.

The final chapter on carcinoma of the vagina includes details of the authors' experience of the recent cases of adenocarcinoma of the vagina in young women whose mothers had received stilboestrol therapy antenatally.

All the Tables and Figures are clear and enhance the text. The references throughout largely reflect the American literature, but the significant contribution from other countries are also given.

This is an excellent book and can be strongly recommended for all post-graduates who have an interest in gynaecological cancer as well as those already involved in this field of work. I have no doubt that this will be regarded as one of the standard books read by the aspiring specialists in gynaecological oncology or radiotherapy.

V. R. Tindall